# Procedure versus process: ethical paradigms and the conduct of qualitative research

**DOI:** 10.1186/1472-6939-13-25

**Published:** 2012-09-27

**Authors:** Kristian Pollock

**Affiliations:** 1 School of Nursing, Midwifery and Physiotherapy, University of Nottingham, Queen’s Medical Centre, Derby Road, Nottingham NG7 2HA, UK

**Keywords:** Qualitative research, Bioethics, Empirical ethics, Micro ethics, Principalism

## Abstract

**Background:**

Research is fundamental to improving the quality of health care. The need for regulation of research is clear. However, the bureaucratic complexity of research governance has raised concerns that the regulatory mechanisms intended to protect participants now threaten to undermine or stifle the research enterprise, especially as this relates to sensitive topics and hard to reach groups.

**Discussion:**

Much criticism of research governance has focused on long delays in obtaining ethical approvals, restrictions imposed on study conduct, and the inappropriateness of evaluating qualitative studies within the methodological and risk assessment frameworks applied to biomedical and clinical research. Less attention has been given to the different epistemologies underlying biomedical and qualitative investigation. The bioethical framework underpinning current regulatory structures is fundamentally at odds with the practice of emergent, negotiated micro-ethics required in qualitative research. The complex and shifting nature of real world settings delivers unanticipated ethical issues and (occasionally) genuine dilemmas which go beyond easy or formulaic ‘procedural’ resolution. This is not to say that qualitative studies are ‘unethical’ but that their ethical nature can only be safeguarded through the practice of ‘micro-ethics’ based on the judgement and integrity of researchers in the field.

**Summary:**

This paper considers the implications of contrasting ethical paradigms for the conduct of qualitative research and the value of ‘empirical ethics’ as a means of liberating qualitative (and other) research from an outmoded and unduly restrictive research governance framework based on abstract prinicipalism, divorced from real world contexts and values.

## Background

At a time when research is promoted as a core activity within national health care systems, it is ironic that the bureaucratic complexity of research governance in the United Kingdom and other developed nations has increased to a point where the regulatory mechanisms intended to protect participants now threaten to undermine and even stifle the research enterprise, especially as this relates to sensitive issues and hard to reach groups. Concerns are mounting that such mechanisms increase the cost, reduce the quality and restrict the scope of research, especially among vulnerable sections of the population who stand to benefit most from the outcome, e.g. older patients and those with dementia, or who are dying. Far from promoting ethical research and protecting research subjects from harm, it is argued that current governance regulations and procedures can actually work against, and undermine, these goals [1-13]. It is further argued that research governance has become onerous to the point of driving research, especially involving pharmaceutical trials, abroad and to areas where research participants may not be adequately protected by regulatory structures and processes [3,14,15]. In relation to qualitative and health services research there is concern that researchers are being deterred from undertaking studies involving sensitive topics and vulnerable populations. As a result research questions are being censored, methods skewed and questions abandoned as a result of the real and anticipated difficulties of securing research governance approvals [8,16-19].

Much critical attention focused on the bureaucratic obstacle of research governance concerns the long delays involved in obtaining research approvals and the bureaucratic restrictions imposed on the conduct of studies [14,15,20,21]. This is particularly burdensome for the substantial and diverse body of research (including qualitative studies and much health service research) which does not involve random control trials (RCTs) or other experimental investigations for which the current regulatory system is principally designed [9,22]. Somewhat paradoxically, recognition of the value of qualitative methodologies in health services research [23-27] parallels the evolution of research governance frameworks which have increasingly constrained their practice. It is argued that although the risks and potential harms of qualitative research are minimal compared to experimental or drugs trials, all studies have been subject to review within the methodological and risk assessment frameworks applied to biomedical and clinical research [4,9,22,28-31]. The need to streamline processes of ethical regulation and review of research has been recognised and processes of consultation and reform are currently in process in countries across Europe, America and Australia [12,32-35] In the UK the Health Research Agency (HRA) was established in 2011 to expedite this process [36-38]. These developments are very welcome. However, simply streamlining the process of ethical review will not solve the problems arising from 1. (unintended) consequences of the legislative frameworks within which Research Ethics Committees and Institutional Review Boards operate [6,39]^a^; and 2. a basic incompatibility between the dominant bioethical framework which underpins the regulatory structures of research ethics and governance and the principles and practice intrinsic to qualitative research^b^[4,9,22,29,30,40-45]. Less attention has been paid to the issues deriving from the different epistemologies which characterize biomedical and qualitative investigations and their implications for conducting research in naturalistic, real world settings. This paper considers the implications of current legal constraints alongside contrasting ethical paradigms for the conduct of qualitative research, particularly in sensitive and difficult areas such as palliative and end of life care, for which such methods of investigation are particularly suitable and the need for further research is pressing.

## Discussion

Concerns about the ethical conduct of research are not a modern preoccupation. Debates within academic disciplines such as anthropology and psychology are longstanding [44]. However, as the bioethical framework underpinning current regulatory structures of ‘procedural ethics’ has achieved dominance within the changing legal and political contexts in which research is undertaken, concerns and practices particular to these other disciplines have been superseded and overwritten. Internal regulation of research governance has been replaced by increasingly centralized control [46]. Similar processes and regulatory structures have developed across Europe, the US, Canada and Australia. Funding and sponsorship of health and, increasingly, social research is dependent on receipt of a ‘favourable opinion’ from an accredited review body (Research Ethics Committee (REC) or Institutional Review Board (IRB)) [47].

### Bioethics

Common (and conflicting) principles underlie the procedurally based bioethical framework characteristic of the systems of ethical appraisal in the UK and elsewhere: 1. autonomy, 2. beneficence (and non-maleficence) and 3. justice [48]. Respect for autonomy requires that research participation must be voluntary and based on fully informed consent, with the right to withdraw at any time. The principle of beneficence/non-maleficence stipulates that research should produce recognizable benefit for both the individuals involved and also the wider community. It should also cause no harm. This extends to the protection of individual privacy and identity through the assurance of confidentiality and anonymity. Justice involves a commitment to the burdens and benefits of research being shared equally throughout all populations and sections of society. It also contains the idea that no section of the population should be excluded from the opportunity to take part in research [49]. The problem is not so much with the principles themselves, but the rigid and formulaic manner of their application within highly bureaucratic and rule based systems of ethical review. In addition, rather than retaining an acknowledgement that the four principles are equal and that balancing may be required where interests conflict [50], autonomy has acquired a uniquely privileged position. This reflects the dominant Western conceptualization of the ‘individual’ as a discrete, self-governing agent (consumer) [42,43,51,52].

### The contingent nature of ethical regulation

Discourses involving ethics invoke transcendent values and absolute principles. However, as evidenced by the proliferation, revision and debates regarding codes and standards the ‘ethical’ frameworks currently regulating research are contingent cultural constructs: the product of particular time, place and competing interests [44,53,54]. Even within the time frame through which current Western bioethics has developed post World War Two, sensibilities have altered in relation to acceptable methods and topics of research and undoubtedly they will change again. The abusive medical experiments of the Nazi regime occurred within a system of regulated research [4,16,54], while the elaboration of guidelines for ethical research has not prevented continuing abuse or mishap, albeit as a (hopefully) infrequent occurrence [55,56]. We look askance at studies undertaken by some previous investigators from the 1960s and 1970s [57-61] through to the present day [62]. Debates continue over ethically acceptable research design and practice among predominantly disadvantaged and impoverished populations of Third World and developing nations [63-66]. The current preoccupation with protection of privacy and the necessity of informed consent dates only from the 1990s [45]. It is evident that similar processes and regulations for ethical review do not produce consistent outcomes, either within or between countries as closely related as members of the European Union [67-70]. In addition, ‘ethical’ principles are interpreted and applied selectively. The principles of the Helsinki Declaration regarding the ethical involvement of human participants in clinical and biomedical research are binding on physicians and should supersede local and national regulations. They supposedly underpin all disciplinary and national research ethical guidelines. However, where conflicting interests arise, as in relation to use of placebos in clinical trials and the conduct of research in countries within the developing world, subsequent revisions of the Declaration have been rejected or omitted by powerful agencies such as the United States Food and Drug Adminstration ( since 2004) and within regulatory frameworks including the European Clinical Trials Directive and the International Conference on Harmonisation of Good Clinical Practice [65,71-74] .

### The case for proportionality

The principle that research should be subject to ethical review and regulation is not at issue. However, it is reasonable that the mechanisms involved should be constructive and proportionate [2-4,13,19,29,75,76]. Biomedical research has the potential to cause harm to participants through known or unanticipated risks of treatment interventions. Bioethical codes are predicated on assumptions of mistrust and hazard: patients need to be protected from risky research which is often linked to powerful commercial interests. Researchers are deemed to require regulation in order to behave ethically [44]. In contrast, the risks of qualitative research are minimal, commensurate with those encountered in everyday life [4,47]. An abiding principal of ethical research in anthropology and other social sciences has been the researchers’ concern and commitment to protect the interests of their respondents. In this context it is the vested interests of powerful commercial and government agencies, not the researcher, that are perceived to constitute the source of potential harm [9,44,46]. The conduct of qualitative health services research has rarely, if ever, been the subject of demonstrable harm or even complaint [4,31]. Indeed, there is evidence that those who take part in qualitative studies often find this to be a positive experience, and are willing to engage in research even when it involves discussion of topics and experiences which they anticipate to be distressing and emotionally challenging. Motivations for such participation may be various and complex. They include curiosity, interest, helpfulness, a chance to receive acknowledgement and reflect on powerful personal experiences and perhaps the possibility of personal benefit from research outcomes and findings. However, a commonly stated motive is altruism, a desire to ‘give back’ and to contribute to work which may benefit others and make a contribution to the wider social good [5,77-94].

### Micro ethics and qualitative research

The bioethical framework of procedural ethics is based on a principle led form of reductive and deductive reasoning which lacks social salience and does not relate to the exigencies of research in real world settings [9,22,29,30,41,42,45,85,95,96]. Corrigan [43] characterizes the outcome of such procedures as ‘empty ethics’. Judgements are based on abstract principles and formulaic rules without knowledge or consideration of context, and in accordance with misplaced assumptions which idealise and reify the process of obtaining informed consent as a ‘rational-technical’ procedure involving ‘fully informed’ and ‘autonomous’ individuals. Information sheets and signed consent forms are widely unread and provide no evidence that respondents have a sufficient understanding of the research they have been invited to take part in; they merely confirm that due process has been observed [85,89]. Indeed, studies of trial participants indicate that respondents’ knowledge and awareness of the nature and purpose of the study is frequently poor [97-99]. A more appropriate approach to protecting the ethical integrity of qualitative research is through recognition of the distinctive epistemological and methodological paradigms involved [30,43,44]. These call for the application of ‘micro’ or ’process’ rather than ‘procedural’ ethics [22,95].

Micro ethics is based on judgement rather than rules, and relies on the cultivation of ‘ethical mindfulness’ on the part of individual researchers. It involves participation of research participants on the basis of trust and motivation during which consent is confirmed as an ongoing process and validated by continuing involvement [43,88,100,101]. Qualitative research depends not just on the willing cooperation of respondents, but on their engagement and collaborative *input*: it is in the process of engagement between researcher and participant(s) that ‘data’ are generated and the research can be accomplished [18,29,42,44,102-104]. The researcher connects with participants, on the basis of reciprocal exchange, through a personal rather than a contractual relationship, and within a relatively even social field [16,105]. The participant needs to get something positive out of the encounter to sustain continuing involvement. This in itself requires that participants are given courtesy and consideration. Since qualitative research rarely involves intervention, research participants are unlikely to feel beholden to the investigator, especially where these are not involved in providing clinical care. In qualitative research participants are constructed as active agents, rather than passive subjects, who bring their own agenda to the encounter, and critically appraise their research experience and involvement. That this may be on the basis of entirely different criteria than those contained within the procedural ethics framework does not mean that their participation is based on bad or unsatisfactory decisions [85,89].

Issues arise because good and innovative qualitative research cannot be squeezed within the constricting frame of bioethics ^c^. By its nature, qualitative research is flexible, emergent and negotiated: the direction of enquiry and even the research question cannot be precisely anticipated in advance, and may alter in response to emerging findings [16,18,29,42,83,102,106]. This is particularly true in relation to ethnographic observation. Long recognized as the gold standard of qualitative research methods, observation has once more come in vogue as a powerful method of exploring naturalistic, real world settings, especially in relation to sensitive topics or with hard to reach and vulnerable populations. However, certain features of qualitative work, including ethnography, conflict directly with the current principles of bioethics which govern medical and health services research.

### Recruitment and access to Personal Identifiable Information (PID)

Current international legislation to protect personal privacy (autonomy) restricts unconsented access to personally identifiable data (PID) [107]. This means that researchers outside patients’ clinical care team (which includes most qualitative investigators) cannot access any personal information –including names, phone numbers and addresses – without participant consent. Legislation also contains enabling measures which allow researcher access to PID where there is no practicable alternative [10,19,45,49,108-111]. However, obtaining the necessary permissions to utilise such measures involves negotiating a complex, bureaucratic and lengthy application process within regulatory systems that are highly conservative and risk averse [9,19]. The prohibition on researcher access to PID derives from legislation concerning privacy rather than ethical principles. Nevertheless, within ethical review of research applications these tend to be conflated, and there is no differentiation between the different kinds and related sensitivities of PID. The restriction on unconsented access to *any kind* of PID is reproduced as a formulaic imperative [10,19,45] Invitations to take part in research are often made by health professionals who would normally have access to PID as direct providers of patient care. This may be reassuring for patients. However, in certain contexts, particularly those involving vulnerability and dependence on critical care provided by clinicians who are also undertaking the research in which the patient is invited to participate, there is a risk of undue influence and coercion [43,85]. Patients may likely feel much freer to reject an invitation without compunction when it is made by an unknown researcher to whom they feel no obligation or desire to please [85,112].

The restriction on access even to names and contact details of prospective participants means that researchers have to rely on health professionals to act as intermediaries in making contact, even when these individuals are not involved directly as investigators. Researchers then have to wait for participants to respond, usually by mail, to an indirect invitation from professional gatekeepers [19,28,113]. The inefficiency of this unwieldy process of requiring patients to opt in, rather than out, of studies produces low rates of recruitment and jeopardises the feasibility and successful outcome of qualitative research. It also hands disproportionate influence over participant selection to health care professionals who may themselves be subjects of investigation. The consequence of prolonged and insufficient recruitment constitutes one of the most frequent and serious challenges to the viability, quality and effectiveness of difficult and costly research [26]. It also undermines the capacity of study findings to be applied directly for patient benefit [3,19,23].

Most of the current or planned reforms to streamline research governance refer specifically to biomedical and epidemiological research and make no reference to qualitative studies. Clarification and revision of the current restrictions on accessing PID for purposes of respondent identification and recruitment should prove helpful to all types of research. Beyond this, however, it is not clear that the proposed reforms will progress a wider awareness and acceptance of the contrasting ethical paradigms which underpin biomedical and qualitative studies.

### Autonomy and informed consent

The primacy accorded to the abstract legal principle of personal ‘autonomy’ within the bioethical framework reflects particular cultural values (individualism and consumerism) of Western industrial societies [45,50,114]. The social sciences offer very different understandings of how individuals position themselves as decision-makers and in relation to others. Decisions are made not by autonomous agents but rather by actors embedded within complex networks of social action and obligation and with awareness of their assessment by, and consequences for, others within both personal and wider social networks [29,42,115]. Bioethics is premised on the research subject as a rational informed self and ignores the relational component of decisions which are made in consultation with, and in awareness of the interests of, others [116]. This includes an assessment of the trustworthiness of science and the social value and benefits of research as well as perceptions and appraisal of the personal consequences of participation [43,85,89,94]. Information provided in the Participant Information Sheet may be understood in ways entirely different to those the researcher intended [112,117]. The symbolic significance of the ritual of informed consent as a cue to the authority and trustworthiness of the researcher may be of greater import than its ostensive significance as a validation of participant understanding of the nature of the study [118]. In practice, information plays a small, and often negligible, part in most participant decisions to take part in research. Such decisions may be impulsive, heuristic and affective, and influenced less by rational deliberation or neutral ‘information’ (however ‘full’) than by discussion and negotiation [85,89,119]. As social actors, individuals are mindful - and accepting – of their obligations, as well as their entitlements. The ‘autonomous individual’ is an ideological construct: an ‘imaginary’. The concept of autonomy may be instrumental within legal, commercial and policy contexts, but it has little salience as a social category. A more useful and appropriate construct in this context is that of ‘agency’. Individual interests are intertwined with those of others, and realized through relationships of obligation and exchange. Consequently, the current preoccupation with autonomy as a singular marker of ‘respect for persons’ and its pre-eminence among the four bioethical principles is unwarranted and calls for revision [50,85,120].

Within the bioethical paradigm, research participation should be voluntary and based on fully informed consent. However, in qualitative research transparency of purpose and of method is not always easy to achieve. This partly derives from the flexibility of qualitative research and its goal of adapting and responding to situations and developments arising in real world settings [83,121,122]. Many observational studies take place in semi-public places (including the internet). It is not practicable to control the movement of people entering and leaving the field, or to ensure that everyone who does so has been adequately informed about the study and given their consent [29,30,41,123]. Researchers simply do not have the control over the research field which is predicated in formal ethical approval. The precise focus of some qualitative studies may deliberately be kept vague. This arises because success may depend on keeping response bias to a minimum or to avoid causing distress in cases where the study focus encompasses an investigation of responses to experiences of diagnosis and prognosis of which participants may be unaware. Even within a single research interview, neither investigator nor participant can fully anticipate the content or outcome of the encounter, or what new topics and insights may emerge. Consequently, it is hard to be highly specific in giving information about the study, or indicating precisely what research participants are signing up to [9,113]. As indicated above, qualitative research engages with participants on the basis of ongoing, process consent. This is tailored, negotiated and reaffirmed throughout the participant’s continuing participation and cooperation in the study. Especially with participants from marginal, stigmatized or deviant populations the requirement for signed consent may be experienced as unwelcome, inappropriate or even coercive, and can impair the development of trust between researcher and participants [9,28,30,85,102,124,125]. Indeed, some people may judge that their interests - and anonymity - are best served and protected by engaging in research with literally ‘no strings attached’.

### Confidentiality

The research enterprise depends on trust based on the promise of confidentiality. This has traditionally been a core commitment of qualitative research. It is a primary means of protecting respondents from any adverse consequences of research participation, and also a precondition of the meaningful exploration of many difficult and sensitive areas of human activity and experience, especially involving marginal and stigmatized social groups [9]. As indicated above, ‘non-maleficence’ is an underpinning principle of research conduct: *participants* should not be harmed through taking part in research. Confidentiality is particularly important when the anonymity of participants cannot always be guaranteed, for example where research involves small or unusual samples, identifiable locations, or ‘elite’ respondents with local or national profiles [9,46,126]. This commitment can give rise to controversial and uncomfortable situations beyond easy compromise or resolution. However, the importance of maintaining trust as an underpinning principle of service delivery is recognised in clinical practice and public health, for example in relation to the concealment of identity of doctors affected by HIV, or HIV patients known to be engaging in unprotected sex [114,127]. It seems reasonable to propose that similar commitments should be honoured in relation to research participation. However, current practices relating to professional ‘duty of care’ have become generalised from health professionals undertaking health services research to all researchers undertaking any kind of research. In principle, and as stipulated in the British Sociological Association and Association of Social Anthropologists statements of ethical guidelines, confidentiality of research participants should only be broken in exceptional circumstances, where issues of public interest are clearly raised [106,128]. In practice, and in view of the uncertainties about current legislation and risk aversion of regulatory bodies, a much more routine and formulaic application has evolved [9,10,45,76,129]. These impose an obligation on researchers to break participant confidentiality in the event that they reveal (what is judged to be) evidence of illegal activity or actual or potential harm to themselves or others [130-132]. The routine requirement to offer participants only qualified or partial confidentiality effectively undermines the basis on which much research involving deviant or marginal populations and sensitive topics may be successfully undertaken. The nature and purpose of research should not be confounded with the practice of health care. This is not to say that circumstances may *never* arise where a researcher feels that it is necessary to break confidentiality. The literature on how researchers have dealt with such dilemmas in practice reveals the nature of ‘ethical’ decisions to be grounded in the particularity of contexts and situations [129,130,133,134]. Researchers are clearly not exempt from the obligation to observe legal and regulatory requirements. However, the occurrence of ethical dilemmas in the field calls for deliberative judgement which can be informed and guided but not resolved by normative procedural rules. Rather than resort to formulas and avoidance, there needs to be acknowledgement and acceptance of the *intrinsically difficult* nature of ethical issues. These do not lend themselves to easy or sometimes even *any* satisfactory solution. In some situations there may not be, self-evidently or unequivocally, ‘a right thing to do’. It may not be possible to support the best interests of all participants. In these situations the researcher can only make the *best* judgement she can, informed by knowledge of the particular context (‘thick ethical description’ [135]) and awareness of the ethical principles and debates relating to each case. In practice, at least within health services research, the occurrence of situations in which breaching confidentiality becomes an issue are extremely rare as, indeed, are other ‘hypotheticals’ (e.g. the prospect of ‘harm’ resulting from qualitative studies) which are nevertheless mandated for inclusion in the standard information sheets supplied to participants.

### Incapacity

The case is clear for relaxing the protectionist and paternalistic stance of current regulatory processes in relation to people taking part in qualitative studies where capacity is not at issue. People can make their own decisions about whether a researcher is trustworthy, if they wish to participate, how they wish to engage in the study and they can withdraw at will. Studies where this is not the case are more problematic, e.g. those involving children, persons with learning disabilities or cognitive impairment. However, further knowledge is badly needed to increase understanding and improve services and care for such vulnerable and frequently under-researched groups. Indeed, it has been argued that current restrictions applying to the inclusion of vulnerable subjects in research constitute actual harm, rather than a benefit or protection, on two counts. Firstly, such groups are denied the benefits which research can confer. Second, in being excluded from the opportunity to take part in research, they are denied the chance to contribute as social agents and to have a voice in the development of policies and services relevant to their condition [4,42,49,100].

The bioethical principle of autonomy requires that research participation should be voluntary, informed and undertaken by persons with capacity to make independent decisions. This formally excludes all persons lacking such capacity from involvement in research. To get around this restriction, a utilitarian fudge can be brought into play. Subject to favourable ethical review, persons lacking capacity may be included in research on the basis of consultation and advice either with a nominated or designated proxy (often a relative) or, where these are not available, an independent advocate [39,100,136,137]. The substituted judgement of such proxy agents may be based on inaccurate or no prior knowledge of the patient or his wishes. These could, in any case, be impossible to determine. It is not usual for individuals to hold and share clearly formulated anticipatory wishes about involvement in research in the event of future incapacity. Even where these are in place, it is a moot point how well future preferences relating to a drastically altered state of cognitive and functional capacity can be anticipated in advance [138]. Consequently, it is not readily apparent how this aspect of the regulations governing inclusion of persons lacking capacity in research serves to protect ‘autonomy’.

In some studies the complex processes involved in obtaining consultee opinions may not pose a problem. In any case, where research involves interventions which are intrusive or entail risk of actual harm, the interests of vulnerable subjects must be safeguarded and participation subject to careful consideration and review. This is the intent and appropriate purpose of current legislation and formal ethical review. In contrast, qualitative studies rarely if ever involve tangible or material threat or harm to participants. However, research occurring in naturalistic settings, particularly where observation is involved, often simply cannot fit within the slow and cumbersome pace of governance bureaucracy: the real world moves too fast. The issue here is the extent to which we are prepared to engage with the ethical dilemmas which may arise in carrying out qualitative research and accept that these may not always be resolvable within the current bioethical framework of research governance. There is often not self- evidently a ‘right’ thing to do, and the most that can be asked is that researchers make the best judgement they can and also that such judgements should be supported by discussion, reflection and knowledge, rather than subject to censure. Within the bioethical framework, individual interests trump those of science and society [45,136]. However, to undertake meaningful qualitative research, a trade off needs sometimes to be made in balancing the interests of the individual against those of the group, the potential intrusion of research against its benefits. This needs to be acknowledged and accepted [19,45,139].

### Ethical mindfulness and the deskilling of researchers

Current governance places the qualitative researcher in an invidious position, caught between formal requirements of ethical approval which do not incorporate any understanding of the pragmatics of field work or the concerns and interests of participants on the ground, and the - equally ‘ethical’ - requirement to maximize the scarce opportunity to deliver best quality research with findings that may offer real impact and benefit to patient care. The standard formulas and oversimplified rules prescribed for research conduct appear to offer solutions whilst eliding the real issues, from the quotidian to the exceptional, to be encountered in real world research. Far from promoting ethical research it has been argued that the rigidity of procedural ethics is deskilling. Rather than encouraging ethical discussion and deliberation - the development of ‘ethical mindfulness’ - procedural codes and regulations merely serve to undercut the researcher’s integrity and judgement [5,16,18,22,28,44,140].

On the ground immediate judgements must often be made about unanticipated events and opportunities which fall outside the formal specification of how a study should be conducted [141]. For example: researchers may inadvertently be presented with personal identifiable data that they are supposed not to access without prior consent. They may have to choose between foregoing an opportunity to obtain data when a respondent volunteers their willingness and availability to conduct an on the spot interview, and adhering to the protocol regarding ‘informed consent’ which requires advance notification and reflection. In observational studies, staff may omit to provide potential respondents with information about the study. The researcher must choose whether it is better to provide this directly to ensure that the information is supplied, or observe the requirement that researchers should not make direct initial contact with participants. In practice it may be impossible to ensure that information has been distributed to patients during admission to a ward. It can be hard to know in advance how many respondents should be recruited. If the specified target is substantially exceeded, or it emerges that representation from different groups is required, data collection must be paused or disrupted pending further approvals of protocol amendments by the REC and NHS research and development departments. This process usually takes several months, with adverse knock on consequences for the completion of the study within schedule. Meanwhile the chance to include specific individuals or groups may have passed, and the opportunity lost. People may offer their participation and cooperation but on their own terms. For example, they may not want to sign consent, or may be willing to talk on the spot but not to arrange a formal meeting in the future. During a scheduled interview in a respondent’s house, a spouse or other person may unexpectedly join in the discussion. The researcher cannot know in advance if they are going to make a brief or more substantial contribution to the interview. Nor can she request that they should leave the room. Gradually, it emerges that the discussion has turned into a joint interview. The researcher is then caught between the formal - but in this case gratuitously disruptive - requirement to go through the process of seeking written consent and risk jeopardizing the interview by violating the social rules of the encounter. Or she can make a pragmatic judgement that both respondent and spouse are comfortable with jointly engaging in the interview and that the spouse’s consent is clearly indicated by his/her participation in the discussion and any questions about the research which commonly arise during and (especially) after the interview. And so on [28,30,95].

In contrast to the formal procedures of bioethical regulation, Pels [44] has called for a ‘morality of negotiation’ and a recognition and tolerance of ‘emergent ethics’ in qualitative research, composed contingently, perhaps not always consistently, but pragmatically, and appropriately for the situation in hand [9,44,104,142]. This shifts the focus away from formal processes of external regulation towards an increased reliance on - and recognition of - the skill, training and *trustworthiness* of individual researchers [18,129]. This approach fits well with the emerging critique of bioethics offered by ‘empirical ethics’. This seeks to remedy the shortcomings of an overly abstract nature of bioethical principalism by engaging with how moral values and dilemmas are embedded and resolved in real world experience and practice. Ethical deliberation would then be informed by an understanding of how and why decisions are made in specific contexts, rather than simply specifying a priori rules for how they *should* be made. For example, increasing evidence that the formal requirement that research participation must be based on ‘informed consent’ is largely unrealisable, and the presumption that research proceeds on this basis is a fiction, should prompt a reappraisal of the current ‘rules’ for negotiating participation. Similarly, a true respect for, and operationalisation of, autonomy would start from an understanding of how research participants understand and value autonomy and modify governance requirements accordingly [99,143-145]. This is not to say that research ethics is then left rudderless on a sea of relativism and descriptive subjectivity, nor that researchers should not be subject to scrutiny and supervision. But it is acknowledged that failure to factor in the context and nature of real world practice and experience results, according to Corrigan’s characterisation, in merely ‘empty ethics’ [43].

### Censoring research

The current difficulty in obtaining ethical approval for qualitative studies of sensitive issues or involving vulnerable populations discourages researchers from attempting to carry out such work. This constitutes a form of censorship, limiting and directing the kinds of research questions that may be asked and how they may be investigated [4,5,9,16,146,147]. In consequence, vulnerable patient groups are excluded from research and the voice which this may give them to apply leverage in improving care [49,148,149]. Misplaced protectionism on the part of ethical committees in closing research to vulnerable groups may constitute a harm rather than intended benefit [4,42].

The conditions which apply to gaining access to personal identifiable data and the requirements for informed consent impose complex constraints on accessing and recruiting participants. Current legislation contains enabling provisions for these restrictions to be overcome in order that research may be undertaken. In practice, however, the conservative and risk aversive culture of ethical review means that the procedures required to obtain such permissions are complex and protracted to the extent of deterring all but the most determined researcher, and perhaps the few who have unlimited time required to pursue the endeavour [11,19,45,69]. The upshot is that qualitative researchers (and others too, but it is qualitative research that I am primarily concerned with here) have become burdened with a system of ethical review that is cumbersome, inappropriate and restrictive to the point that it is making research, particularly in relation to vulnerable populations and sensitive topics, increasingly hard and sometimes even impossible. The need for some form of external regulation and ethical review is not contested, but this should be proportionate to the risks involved and conducted with sufficient expertise. The potential harms of qualitative research should be acknowledged, for example: intrusion into personal privacy, arousal of distress, embarrassment, or breaches in confidentiality. However, these are rare, relatively minor and commensurate with the normal vagaries of everyday life. The literature reveals no known or recorded instance of complaint or harm, and suggests that many respondents find research participation to be a positive experience even when this involves discussion of topics that may be difficult and distressing. Much research is no more intrusive or invasive of personal privacy than routine clinical audit or service evaluation, but it is treated radically differently [19]. This suggests that the function of current governance arrangements is to control and discipline, rather than promote and enable, the research effort [4,5,8,9].

### Rebalancing beneficence autonomy and justice

Given the promotion of research as a core activity and political priority within the health services of developed nations [37,150-152] the widespread public acceptance of research, and their willingness to participate in contribution to a public good, it is reasonable to revise current conventions which routinely assign autonomy pre-eminence over other ethical principles. Perhaps it is time to move the bar a little more towards the ‘community’ rather than the ‘individual’ end of the interests spectrum, from the current privileging of ‘autonomy’ towards a greater emphasis on ‘beneficence’ and ‘justice’ [19,45,49,139,153,154]. Patients who benefit from treatment within the NHS or other health care systems can expect to find research being carried on as a routine activity, and that it is reasonable to be approached with an invitation to participate, perhaps even to presume acceptance rather than rejection, at least in relation to non-intervention research involving minimal risk [19,45,49,153]. This is, of course, always on the understanding that participation is voluntary and can be readily withdrawn. Accredited researchers working on approved studies and bound by the same standards of confidentiality as clinicians could (as formerly) be permitted access to patient data to the extent that this is required to conduct their research [75]. It is reasonable to propose that in relation at least to non-invasive research (qualitative and health service studies) a shift from in principle opt in to opt out from research would be acceptable. In addition, patients could be asked to indicate their willingness to be approached about research as a routine part of GP registration or hospital care. Such changes depend on public acceptance and support, and much further involvement of the public in discussion and debate about the social value of research and how it should be conducted is required [10,19,154].

## Summary and conclusion

Ethical regulation has become increasingly complex and restrictive without any evidence that the current machinery has improved the quality of qualitative research or protected research participants from substantive harm. However, it is clear that qualitative research does not fit within the current bioethical framework of ethical regulation which was designed to protect research participants from the very real harms which could be involved in taking part in experimental studies including commercial drugs trials. The need for ethical review and regulation is not at issue. However, this must be appropriate and proportionate to the risks involved. Existing regulatory frameworks disenfranchise qualitative researchers by preventing them from carrying out flexible, responsive research according to accepted best practice within their academic disciplines. They force adherence to a bureaucratic system of procedural ethics which is ill suited to the nature of the research and which, in the worst case, works ‘unethically’ to compromise the quality and reach – and consequently also the value - of the findings. The greatest protection for participants in qualitative studies is for adequately skilled and experienced researchers to conduct and supervise research. Researchers should, of course, be accountable for their actions and work within institutional systems of supervision and support. However, moving responsibility from an over-reliance on external audit towards a greater degree of internal accreditation and review would exert powerful leverage in increasing the quality and integrity of qualitative research.

The rule-based procedural ethics which dominates current regulatory structures is fundamentally at odds with the practice of emergent and continually negotiated processes of ‘micro ethics’ required to undertake qualitative research which is both feasible and maintains integrity. The complex and shifting nature of real world settings (occasionally) deliver substantial ethical issues which may not lend themselves to easy resolution, but this is the exception, rather than the norm. Procedural ethics cannot address the specific ethical dilemmas that arise in the real world settings of qualitative investigation. This is not to say that qualitative studies are ‘unethical’, but that their ethical conduct cannot be safeguarded by adherence to a rigidly pre-determined protocol. Rather, such safeguarding depends on the exercise of ongoing judgement and personal integrity of researchers in the field. Bioethics prioritises procedures and formulas (giving out information, obtaining a signed consent form) over process (the quality of the discussion about the research, establishing the degree of participants’ understanding and willingness to take part). Qualitative studies need to be evaluated within a different set of criteria than that which is applied within the bioethical framework which dominates current systems of ethical review and by reviewers who have a proper understanding of the methodologies to be employed and the realities of conducting sensitive research in naturalistic settings. Empirical ethics is proposed as an approach to counteract the abstract formalism of procedural bioethics, and improve the relevance and quality of research governance through an understanding of the context of participants’ decision making in real world settings, and the values and reasoning underpinning these. It is to be hoped that current national and international proposals to streamline processes of ethical review will expedite these changes and stimulate a review and clarification of current legislation. There is also a need to actively engage the public in dialogue about the role and value of research and how they may be willing to support this as a collective social endeavour. If the public is to benefit from research then it is incumbent that the effort and resources invested – especially in difficult and sensitive areas of investigation – are used to best effect. Indeed, clear ethical issues are involved in failing to do so.

## Endnotes

^a^In the UK, this includes the Mental Capacity Act, 2005; Data Protection Act, 1998, Freedom of Information Act, 2000 and Health and Social Care Act, 2001. Internationally, such protective legislation has become widespread, e.g. European Data Protection Directive (1995), Clinical Trials Directive, 2001, NIST *Guide to Protecting theConfidentiality of Personally IdentifiableInformation* (USA), Privacy Act, 1988 (Australia), Privacy Act, 1983 (Canada).

^b^The Advance Notice of Proposed Rulemaking (ANPRM) under consideration in the US is unusual in proposing changes based on assessment of risk and proportionality which, if implemented, should prove highly enabling for qualitative research 12. Emanuel EJ, Menikoff J: Reforming the Regulations Governing Research with Human Subjects. *New England Journal ofMedicine* 2011, 365:1145–1150, 40. Human Subject Research Protections: Enhancing Protections for Research Subjects and Reducing Burden, Delay, and Ambiguity for Investigators, FAQs [ http://www.hhs.gov/ohrp/index.html; http://www.hhs.gov/ohrp/humansubjects/anprmqanda.html/].

^c^The bioethical framework underpinning research governance also causes problems for researchers engaged in biomedical and other kinds of research, but consideration of these is outside the scope of the present paper.

## Competing interests

The authors declare that they have no competing interests.

## Pre-publication history

The pre-publication history for this paper can be accessed here:

http://www.biomedcentral.com/1472-6939/13/25/prepub
